# Use of healthcare administrative claims data in observational studies of antirheumatic drug effects on pregnancy outcomes: A scoping review

**DOI:** 10.1371/journal.pone.0319703

**Published:** 2025-03-31

**Authors:** Shenthuraan Tharmarajah, Araniy Santhireswaran, Yasmeen Ameeriar, Lisa M. McCarthy, Dharini Mahendira, Howard Berger, Mina Tadrous, Sara J. T. Guilcher

**Affiliations:** 1 Department of Pharmaceutical Sciences, Leslie Dan Faculty of Pharmacy, University of Toronto, Toronto, Ontario, Canada; 2 Institute for Better Health, Trillium Health Partners, Mississauga, Ontario, Canada; 3 Women’s College Research Institute, Women’s College Hospital, Toronto, Ontario, Canada; 4 Division of Rheumatology, St. Michael’s Hospital, Toronto, Ontario, Canada; 5 Department of Medicine, University of Toronto, Toronto, Ontario, Canada; 6 Division of Maternal Fetal Medicine and Obstetric Ultrasound, St. Michael’s Hospital, Toronto, Ontario, Canada; 7 Department of Obstetrics and Gynecology, University of Toronto, Toronto, Ontario, Canada; 8 Department of Physical Therapy, Temerty Faculty of Medicine, University of Toronto, Toronto, Ontario, Canada; Osaka University of Pharmaceutical Sciences, JAPAN

## Abstract

The safety of antirheumatic drugs in pregnancy and their impact on maternal and neonatal outcomes are understudied. Despite pregnant individuals being excluded from clinical trials, their continued use of medications raises the importance of addressing knowledge gaps regarding safety and impact on outcomes. A scoping review was conducted following JBI methodology and PRISMA reporting guidelines to describe how antirheumatic drugs and associated adverse pregnancy outcomes have been investigated in observational studies using claims data. Electronic databases (MEDLINE (Ovid), Embase (Ovid), and CINAHL (EBSCO)) and grey literature were searched for observational studies using claims data to evaluate antirheumatic drug effects on pregnancy outcomes in individuals with rheumatic diseases. Of 4,325 articles identified, 38 eligible articles were included. The effects of conventional synthetic disease-modifying antirheumatic drugs (n =  37, 97.4%) and tumor necrosis factor inhibitor biological agents (n =  23, 60.5%) were extensively reported. Preterm birth (n =  25, 65.8%), preeclampsia (n =  17, 44.7%), stillbirth (n =  17, 44.7%), caesarean delivery (n =  16, 42.1%), and congenital anomalies (n =  14, 36.8%) were the most reported adverse pregnancy outcomes. Of 14 studies reporting congenital anomalies, 12 (85.7%) specified ICD codes and 4 (28.6%) specified validated definitions for identification in claims data, the most of any reported adverse pregnancy outcome. We found considerable ambiguity and heterogeneity in adverse pregnancy outcome definitions in claims data. There is a need for greater transparency and consistency in outcome reporting in observational studies using claims data.

**Protocol registration details:** OSF, https://osf.io/5e6tp

## Introduction

Pregnancies in individuals affected by rheumatic diseases such as rheumatoid arthritis (RA), systemic lupus erythematosus (SLE), psoriatic arthritis (PsA), and ankylosing spondylitis (AS) are complicated by the chronic pro-inflammatory state and ensuing systemic effects that characterize these conditions. As a result, these pregnancies warrant close monitoring due to being at an increased risk for maternal and neonatal complications, including pre-eclampsia, stillbirth and miscarriage, preterm birth, low birth weight and small-for-gestational age, low Apgar score, and congenital anomalies [1–3]. These adverse pregnancy outcomes (APOs) are mediated by maternal disease activity, including flares and remission [4–6]. As a result, optimizing pregnancy outcomes is contingent upon the adequate control of disease activity through medications that are safe and compatible with pregnancy [7–9].

Rheumatic diseases are typically treated with disease modifying antirheumatic drugs (DMARDs), but there remain substantial knowledge gaps regarding the safety of their use in the setting of pregnancy, especially for more recently developed drugs like biological DMARDs [10]. Further, due to the exclusion of pregnant individuals from drug trials, clinicians must often extrapolate data from animal studies and non-pregnant individuals to make critical decisions regarding the care of pregnant individuals with rheumatic diseases such as RA, SLE, PsA, and AS [11]. However, since most of these patients continue to be maintained on DMARD therapy during pregnancy, these gaps can be addressed by leveraging routinely collected population-based healthcare administrative claims (hereafter “claims”) data [12]. For example, claims data on pregnancy outcomes can be linked to other claims databases capturing patient hospitalizations, outpatient doctor visits, emergency department visits, and pharmacy dispensations. Repurposing claims data for clinical and health services research in this manner provides an indispensable and effective tool for evaluating DMARD safety during pregnancy in the real world, despite the unavailability of clinical trial data [13].

In an attempt to use such real-world data to fill current knowledge gaps regarding DMARD safety in pregnancy, we focused this scoping review on observational studies that used claims data to examine DMARD effects on pregnancy outcomes. Although past systematic and scoping reviews [14–17] in this area have summarized data from clinical trials as well as observational study designs, there have not been any such reviews of observational studies specifically using claims data or other types of real-world data (e.g., electronic health records, disease registries, patient-generated data). Therefore, we conducted this scoping review to describe the characteristics of such studies and elucidate methodologically significant knowledge gaps in pregnancy outcome studies. Specifically, we aimed to identify which DMARDs used to treat pregnant individuals with RA, SLE, PsA, and/or AS have been reported in observational studies using claims data; which APOs have been reported in these populations; and how APOs have been defined and identified in claims data using ICD codes. These findings will expand our current understanding of how DMARD effects on pregnancy outcomes in patients with rheumatic diseases have been investigated and can be further refined to inform future studies on this population and generate real-world evidence for DMARD use during pregnancy.

## Methods

This scoping review was conducted in accordance with the JBI methodology for scoping reviews [18] and the Preferred Reporting Items for Systematic Reviews and Meta-Analyses extension for Scoping Reviews (PRISMA-ScR) reporting guidelines [19]. This scoping review was registered on Open Science Framework and followed an *a priori* protocol [20], in which detailed methods have been described. To summarize, we assessed observational studies (e.g., cohort, case-control study designs) using claims data to study antirheumatic drug effects on pregnancy outcomes (Table 1). We included all drugs that indicate APOs within ATC classes M01 (anti-inflammatory and antirheumatic products) and L04 (immunosuppressants). Studies were excluded if full-text articles were not available, study designs were ineligible, individuals with RA, SLE, PsA, and/or AS were not included in the study population, DMARDs were not the primary exposure, pregnancy outcomes were not a study outcome, and/or claims data were not used to define pregnancy outcomes.

**Table 1:** Eligibility criteria.

Component	Description
Participants	Adult pregnant individuals (≥ 18 years) with RA, SLE, PsA, and/or AS receiving csDMARDs and/or bDMARDs
Concepts	csDMARDs include hydroxychloroquine, azathioprine, sulfasalazine, cyclosporine, tacrolimus, methotrexate, leflunomide, mycophenolic acid/ mycophenolate mofetil, mercaptopurine, cyclophosphamide, and colchicine bDMARDs include adalimumab, certolizumab-pegol, etanercept, golimumab, infliximab, rituximab, abatacept, belimumab, anakinra, canakinumab, tocilizumab, ustekinumab, secukinumab, and ixekizumab Observational studies examining antirheumatic drug effects on pregnancy outcomes
Context	APOs must be defined using claims data involving International Classification of Diseases, Ninth and Tenth Revisions (ICD-9 and ICD-10) diagnostic and/or procedural codes Studies conducted using claims data from all regions and settings Studies published in any language
Types of sources	Published observational studies (e.g., cohort, case-control study designs) Unpublished observational studies and reports from relevant healthcare and government organization websites considered as sources of grey literature

We searched for studies published in any language from database inception to December 10, 2022 in MEDLINE (Ovid), Embase (Ovid), and CINAHL (EBSCO). An initial search was conducted on December 10, 2022 and updated on July 31, 2024 to include papers published since. The full search strategies are provided in the published protocol [20]. In addition, we searched OpenGrey, Health Services Research Projects in Progress, the World Health Organization Library, and Google Scholar using modified search terms for sources of unpublished grey literature. We also hand-searched clinical guidelines and policy statements by the American College of Rheumatology, British Society for Rheumatology, and European Alliance of Associations for Rheumatology for potential sources. Lastly, we screened the reference lists of eligible articles for additional data sources.

Literature searching, title and abstract screening, and full text review of eligible articles were independently completed by two authors (ST, AS). Two authors (ST, YA) then independently extracted data from eligible articles, including publication information, DMARDs studied (e.g., hydroxychloroquine), APOs reported (e.g., preterm delivery), number and types of ICD codes (diagnostic and/or procedural) used to define APOs, and citations for validation of coding definitions used. This review did not consider the effects of targeted synthetic DMARDs as outcomes, including those of small molecule Janus kinase inhibitors (e.g., tofacitinib, upadacitinib, and baricitinib), which are generally contraindicated in pregnancy due to a lack of sufficient evidence of no harm [7–9]. Additionally, pre-existing maternal comorbidities such as pre-existing hypertension were not considered to be APOs, and therefore their details were not considered for data extraction. Finally, other coding systems such as Healthcare Common Procedure Coding System (HCPCS), Current Procedural Terminology (CPT), and Canadian Classification of Health Interventions (CCI) were not considered for data extraction on procedural codes. However, the few papers that did use these other coding systems for identifying procedures also used ICD codes, so they were still included in the review and captured in data extraction. Further, for procedures like caesarean delivery, the procedural codes used were often not reported in the paper, often using other coding systems. Any disagreements between the reviewers regarding article screening or data extraction were resolved through discussion or by a third reviewer (MT).

Study characteristics, reported DMARDs, and reported APOs and their coding definitions for identification in claims data were summarized in tabular form. We also summarized the top five most reported APOs. Additionally, we considered an APO definition to be “well-defined” if it included 1) the specific codes or the number and types (diagnostic or procedural) of ICD codes used, 2) the locations of health services provided, and 3) the time period in which they were provided. We further stratified results by APO and whether studies indicated specific ICD codes used and citations for validation of coding definitions used.

## Results

### Study inclusion

**Fig 1:**
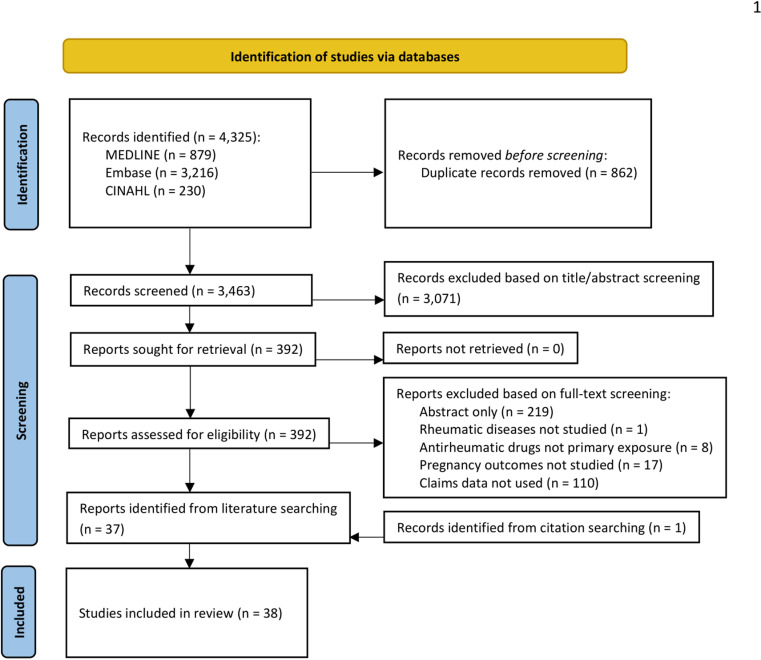
PRISMA flow diagram of study inclusion.

The results of the search and the study inclusion process are presented in a PRISMA flow diagram below (Fig 1) [21]. Of 4,325 unique publications identified based on the relevance of their titles and abstracts, 392 full-text articles were screened, and 38 publications were included: 37 from our initial search and 1 from screening the reference lists of articles identified from our initial search (Fig 1). We also searched through grey literature and identified 80 additional sources, but none met our inclusion criteria.

### Characteristics of included studies

Studies were conducted in 11 countries across 3 regions: North America (n =  16, 42.1%), Europe (n =  18, 47.4%), and Asia (n =  4, 10.5%). In North America, 8 (21.1%) studies were conducted in Canada, with another 8 (21.1%) conducted in the United States. In Europe, studies were often conducted including data from multiple countries, with 10 (26.3%) studies including data from Sweden, 6 (15.8%) including data from Denmark, 2 (5.3%) studies each including data from Norway, Finland, and Italy, and 1 (2.6%) study each including data from Iceland and Germany. In Asia, 3 (7.9%) studies were conducted in Korea, while 1 (3.3%) study was conducted in Taiwan (see S1 Table).

In terms of observational study designs used, there were 32 (84.2%) cohort studies and 6 (15.8%) case-control studies. Regarding the prevalent rheumatic conditions of interest (RA, SLE, PsA, and/or AS), RA was considered in 23 (60.5%) studies, SLE in 19 (50.0%) studies, PsA in 16 (42.1%) studies, and AS in 17 (44.7%) studies. There was 1 (2.6%) study that indicated “rheumatic diseases” but did not specify which conditions.

### Reported antirheumatic drug therapies

The majority of studies (n =  26, 68.4%) examined the effects of both conventional synthetic DMARDs (csDMARDs) and biological DMARDs (bDMARDs), while 11 (28.9%) studies examined the effects of csDMARDs only and 1 (2.6%) study examined the effects of bDMARDs only. Within the 27 (71.1%) studies that examined the effects of bDMARDs, 15 (39.5%) studies examined the effects of both tumor necrosis factor inhibitors (TNFis) and non-TNFis, 8 (21.1%) studies examined the effects of TNFis only, and 4 (10.5%) studies did not specify which bDMARDs were considered. Likewise, 4 (10.5%) studies did not specify which csDMARDs were considered (see S2 Table).

Regarding specific csDMARDs considered to be safe and compatible with pregnancy [7–9], hydroxychloroquine (n =  30, 78.9%), azathioprine (n =  26, 68.4%), sulfasalazine (n =  24, 63.2%), and cyclosporine (n =  19, 50.0%) were most commonly reported. Tacrolimus was also reported in 7 (18.4%) studies, while 1 (2.6%) study considered colchicine. Of note, the use and discontinuation of methotrexate (n =  25, 65.8%), leflunomide (n =  20, 52.6%), mycophenolic acid/ mycophenolate mofetil (n =  14, 36.8%), and cyclophosphamide (n =  5, 13.2%), which are known to be teratogenic and therefore contraindicated during pregnancy, were also widely reported [7–9].

Regarding specific bDMARDs, etanercept (n =  22, 57.9%), adalimumab (n =  21, 55.3%), infliximab (n =  21, 55.3%), golimumab (n =  18, 47.4%), and certolizumab-pegol (n =  15, 39.5%) were the most reported bDMARDs. Of note, these 5 drugs comprise the TNFi category of bDMARDs. Of non-TNFi biological agents, abatacept (n =  12, 31.6%), tocilizumab (n =  10, 26.3%), rituximab (n =  10, 26.3%), anakinra (n =  9, 23.7%), and ustekinumab (n =  7, 18.4%) were most reported. Additionally, belimumab was reported in 5 (13.2%) studies, while secukinumab was reported in 3 (7.9%) studies. Ixekizumab was reported in 1 (2.6%) study, and no studies considered canakinumab.

### Reported adverse pregnancy outcomes and coding definitions

A wide range of APOs were reported across the included studies (see S3 Table). Preterm birth (n =  25, 65.8%) was the single most reported APO, followed by preeclampsia (n =  17, 44.7%), stillbirth (n =  17, 44.7%), caesarean delivery (n =  16, 42.1%), and congenital anomalies (n =  14, 36.8%). The ICD-9 and/or ICD-10 codes used to identify the most reported APOs in claims data are shown in S4 Table. Although preterm birth was the most reported APO overall and reported in 25 studies, only 3 (12.0%) of these 25 studies specified ICD codes, 1 (4.0%) of which also specified a validated definition for identifying preterm birth in claims data (see Table 2). Instead, 19 (76.0%) of the 25 studies defined preterm birth as delivery at less than 37 weeks of gestation, of which 8 (32.0%) studies further stratified preterm birth as moderately preterm (<37 weeks) and severely preterm (<32 or < 34 weeks). The remaining 6 (24.0%) of 25 studies did not specify definitions for preterm birth based on gestational age.

**Table 2:** Summary of top five most reported APOs and their definitions.

APOs	Studies, n	Studies specifying ICD codes, n (%)	Studies specifying validated definitions, n (%)
Preterm birth	25	3 (12.0)	1 (4.0)
Preeclampsia	17	12 (70.6)	4 (23.5)
Stillbirth	17	5 (29.4)	2 (11.8)
Caesarean delivery	16	3 (18.8)	0 (0.0)
Congenital anomalies	14	12 (85.7)	4 (28.6)

In contrast, of the 14 studies that reported congenital anomalies, 12 (85.7%) specified ICD codes and 4 (28.6%) further specified validated definitions that included 1) the specific codes or the number and types (diagnostic or procedural) of ICD codes used, 2) the locations of health services provided, and 3) the time period in which they were provided, more than for any other commonly reported APO (see Table 2). This was followed by preeclampsia, which was reported in 17 studies and specified ICD codes for in 12 (70.6%) and validated definitions for in 4 (23.5%). For stillbirth, 5 studies specified ICD codes, of which 2 studies also specified a validated definition. Finally, 3 studies specified ICD codes for caesarean delivery, with none specifying a validated coding definition for identifying whether caesarean delivery was electively planned beforehand or indicated during labor as an emergency measure.

## Discussion

In this scoping review, we identified and summarized literature exploring antirheumatic drug effects on pregnancy outcomes, as reported in population-based observational studies leveraging healthcare administrative claims data. Across our review of 38 eligible studies, we found that the effects of csDMARDs and TNFi bDMARDs on pregnancy outcomes have been commonly reported. A wide range of APOs have also been reported in pregnant individuals with RA, SLE, PsA and/or AS, with preterm birth, preeclampsia, stillbirth, caesarean delivery, and congenital anomalies being the most reported APOs. At the same time, the ICD coding definitions for identifying these APOs in claims data have not been well-defined or validated in the current literature.

The widespread reporting of csDMARDs is not surprising, as current clinical guidelines recommend csDMARDs as the first-line treatment for most rheumatic diseases [22,23]. Furthermore, many csDMARDs have been widely available and used for several decades, long enough to accumulate sufficient pregnancy safety data [24]. For example, methotrexate, leflunomide, mycophenolate mofetil, and cyclophosphamide are known to be teratogenic, with users of reproductive age requiring appropriate contraception [7–9]. Those actively planning a pregnancy are strongly advised to discontinue them at least three months prior to conception and switch to csDMARDs more compatible with pregnancy, such as hydroxychloroquine, azathioprine, sulfasalazine, and cyclosporine [7]. While several reviewed studies reported on teratogenic csDMARDs, the actual number of users in each study was low. For example, Viktil et al. found that methotrexate had been dispensed to a total of 8 pregnant individuals, with 7 receiving methotrexate during the 3 months prior to conception, 2 during the first trimester, and 1 also during the second trimester. Similarly, leflunomide had been dispensed to a total of 2 pregnant individuals, with 1 receiving leflunomide during the 3 months prior to conception and 2 during the first trimester [25]. This suggests that either these pregnancies may not have been planned or these patients may not have been aware for some time that they were pregnant [26–28].

In contrast, most bDMARDs still lack sufficient evidence of no harm due to their more recent development and are currently indicated for use only in the absence of more appropriate alternatives [7–9]. There is some evidence to support the use of TNFis (etarncept, adalimumab, infliximab, golimumab, and certolizumab-pegol), particularly certolizumab-pegol due to its large molecular weight and consequent minimal placental transfer [14,29–31]. Accordingly, TNFis were the most commonly reported bDMARDs, while the non-TNFi biological agents were not as widely reported. More observational studies reporting on bMARDs in general, and non-TNFi biological agents in particular, are needed to better understand and establish real-world pregnancy safety data for this drug class.

Although a wide range of APOs were reported, these outcomes were studied and reported to varying degrees. As a group, non-live birth outcomes like stillbirth (n =  17, 44.7%), miscarriage (n =  10, 26.3%), elective termination (n =  10, 26.3%), and molar and ectopic pregnancy (n =  6, 15.8%) were the most frequently reported APOs. The hypertensive disorders of pregnancy including preeclampsia (n =  17, 44.7%), eclampsia (n =  7, 18.4%), and gestational hypertension (n =  11, 28.9%) were also broadly reported as a group. In terms of individual APOs, preterm birth, preeclampsia, stillbirth, caesarean delivery, and congenital anomalies were the most reported APOs. On the other hand, few studies reported on bleeding complications like placenta previa and placental abruption (n =  1, 2.6%), threatened miscarriage (n =  2, 5.3%), and hemorrhage (n =  3, 7.9%). Additionally, complications during labor (n =  4, 10.5%) and low Apgar score (n =  5, 13.2%) were also not frequently reported, suggesting that they may not be adequately captured systematically. Even better reported APOs like congenital anomalies may be actually underreported due to elective and therapeutic termination of pregnancy [32]. Future observational studies should endeavor to investigate these lesser reported yet crucial APOs as well as further enhance our understanding of commonly reported APOs. Additionally, singular measures such as preeclampsia, preterm birth, and small-for-gestational age are often not sensitive enough to differentiate pregnant individuals and infants with severe maternal morbidity and severe neonatal morbidity [33]. Therefore, future observational studies should also aim to measure and report these composite outcomes using claims data.

Further, few studies clearly indicated how these APOs were defined and identified in claims data. Congenital anomalies were reported in 14 studies, of which only 5 provided definitions that included 1) the specific codes or the number and types (diagnostic or procedural) of ICD codes used, 2) the locations of health services provided, and 3) the time period in which they were provided. Of the 4 studies that also cited validations for how congenital anomalies were defined, 2 cited the same validation study by Blais et al. [34], which investigated the validity of congenital malformation diagnostic codes in Quebec’s administrative databases. However, only 1 study used the full definition of congenital anomalies as described in the original validation article [34], while the other study used only the portion of the definition that describes congenital heart defects. Of the remaining 2 studies, 1 cited another validation study [35], while the authors of the other study performed their own validation against patient medical charts. This finding reflects substantial heterogeneity among claims definitions of congenital anomalies, making comparisons of drug safety and effectiveness between claims studies difficult. For other APOs like preterm birth, stillbirth, and caesarean delivery, very few studies specified the ICD codes or definitions used to identify these APOs in claims data. This lack of transparency and resulting ambiguity in reporting similarly make reproducibility and comparisons of drug safety and effectiveness between claims studies a pressing challenge [36]. We strongly encourage future claims studies to be more transparent in their reporting of APO definitions, so that it may be more feasible to compare definitions across studies.

Our review has several notable strengths. Firstly, it utilized the robust JBI methodology for scoping reviews and was conducted in accordance with an a priori published protocol [20]. Additionally, to our knowledge, our review is the first study to document APO definitions reported in observational studies using claims data to investigate antirheumatic drug use during pregnancy, while previous systematic and scoping reviews on this topic have examined data from randomized clinical trials and other study designs [14–17]. Further, to minimize the risk of our search strategies potentially not including all terms relevant to these concepts, we consulted with a health science librarian and content experts (DM, HB) on our team in rheumatology, pregnancy, and related therapy management to ensure that subject headings and key words were comprehensive. Lastly, we did not limit our review by region, setting, or language and were therefore able to include studies from around the world.

At the same time, our review also has a few limitations that warrant discussion. Our exclusion of conference abstracts and proceedings may have omitted findings from some studies conducted in other settings, but these sources would not have fit the purpose of this review given the lack of data that we could have extracted from them. Further, it is possible that we may have missed some articles in our review of grey literature. Importantly, we acknowledge that there may have been limitations in our interpretation of APO definitions due to the ambiguity in reporting. Although we described APO definitions as they were reported by the authors of each paper, we considered a definition to be “well-defined” only if it included 1) the specific codes or the number and types (diagnostic or procedural) of ICD codes used, 2) the locations of health services provided, and 3) the time period in which they were provided. Therefore, while many studies indicated “inpatient and outpatient diagnostic codes” to identify APOs, there were no specifications regarding the exact number of codes or the time period in which they were provided. As a result, although such definitions may in fact have been effective at identifying APOs, the manner in which they were reported did not allow us to assess their rigor, and therefore we did not consider them to be “well-defined.” Given this ambiguity, we recommend that future observational studies using claims data make detailed APO coding definitions available in text or supplemental material as well as align with reporting standards for observational studies [37–40]. Additionally, since our review was not restricted by region to North America alone, we chose to consider only ICD codes and definitions and did not consider other coding systems such as HCPCS, CPT, and CCI for data extraction on procedural codes. However, the few papers that used these other coding systems for identifying procedures also used ICD codes, so they were still included in the review and captured in data extraction. Therefore, we do not believe the anticipated impact of not including these coding systems on our findings to be significant. However, this may also have been a potential limitation, especially in the case of certain outcomes like caesarean delivery, which is in itself a procedure.

Our scoping review focused on which DMARDs and APOs have been studied using claims data and how those APOs have been defined using coding algorithms. At the same time, there are other very relevant aspects of pregnancy studies, such as how pregnancies were identified in individual databases, how important dates (e.g., conception, last menstrual period, delivery) were estimated, how diseases were defined using coding algorithms, and how long the lookback windows for drug exposure and comorbidities were. Future literature reviews on the use of claims data in observational pregnancy studies should explore and summarize these pertinent components of using claims data to study pregnancies. Similarly, future literature reviews should also consider the statistical methods applied in the study of drug exposures on APOs, as well as which comparator groups were used and whether analyses were adjusted. Finally, future work should assess the quality, rigor, and significance of findings from observational studies of pregnancy using claims data to ensure generalizability to different pregnant populations and inform clinical drug prescribing practices.

Claims data offer a valuable opportunity for pharmacoepidemiologic studies to generate much-needed real-world evidence for vulnerable yet historically understudied populations like pregnant individuals with rheumatic diseases and other chronic conditions [41]. Obtaining drug dispensation data before and during pregnancy circumvents recall bias that may be present in patient surveys. At the same time, challenges in using claims data to study pregnancy outcomes include coding misclassification, billing errors, and “rule-out” diagnoses [42]. Further, in-hospital drug dispensations would also not be captured, and a filled prescription also does not necessarily entail patient adherence. Therefore, drug use may not be accurately reflected in claims data. Similarly, details on maternal disease activity and severity, date of conception, and anatomically relevant information are not available in claims data, often necessitating linkage with registry data on pregnancy and/or diseases. Nonetheless, despite these limitations and challenges, claims data pose a methodological advantage and alternative in the study of pregnant individuals, who are frequently excluded from clinical trials.

In our review, we found substantial ambiguity and heterogeneity in the reporting of methods used to identify APOs, and while our review focused on the risk of APOs following DMARD use during pregnancy, similar discrepancies may be found in studies examining the risk of APOs following prenatal exposures to other drugs as well (e.g., antihypertensives). Study results across various settings and study designs are most easily comparable when they use consistent APO definitions that are also made readily transparent [36]. Therefore, APO definitions should be reported in a clearer and more consistent manner in future observational studies using claims data. Specifically, we recommend that for each APO, future studies should at a minimum report 1) the specific ICD codes or the number and types (diagnostic or procedural) of ICD codes used, 2) the locations of health services provided, and 3) the time period in which they were provided. Finally, the vast variations in APO definitions seen in our findings suggest that more studies are needed to develop and validate definitions to identify specific APOs in claims data. We hope that these findings will contribute to our understanding of how DMARD effects on pregnancy outcomes are investigated in observational studies using claims data as well as inform future such studies to generate real-world evidence for the safe and effective use of DMARDs during pregnancy.

## Supporting information

S1 Table Characteristics of included studies (N =  38). (PDF)

S2 Table Summary of DMARDs reported in included studies (N =  38). (PDF)

S3 Table Summary of APOs reported in included studies (N =  38). (PDF)

S4 Table ICD-9 and/or ICD-10 codes used to identify most reported APOs in claims data. (PDF)

S1 Checklist Completed Preferred Reporting Items for Systematic reviews and Meta-Analyses extension for Scoping Reviews (PRISMA-ScR) checklist. (PDF)
