# Supplementary material for: Use of healthcare administrative claims data in observational studies of antirheumatic drug effects on pregnancy outcomes: A scoping review
Source: PLoS One. 2025 Mar 31;20(3):e0319703. doi: 10.1371/journal.pone.0319703 (PMC11957274; doi:10.1371/journal.pone.0319703)
Supplement: S1 Table — (PDF) [file pone.0319703.s001.pdf]

S1 Table.

ID	Citation	Country	Study design	Objective(s)	Data sources	Diseases	Medications	APOs
1	Kristjansdottir SR, Steingrimsdottir T, Grondal G, Bjarnadóttir RI, Einarsdottir K, Gudbjornsson B. [Pregnancy outcomes in Icelandic female patients with inflammatory arthritides. Nationwide results from the ICEBIO and the Icelandic Medical Birth Register]. Laeknabladid. 2019;105(6):267-275. Icelandic.	Iceland	Cohort	to investigate the possible effects of severe arthritis on pregnancies and births of Icelandic women	Icelandic medical registers	RA, PsA, AS	adalimumab, etanercept, golimumab, infliximab	preterm birth, caesarean delivery, low Apgar score, low birth weight, high birth weight
2	Bröms G, Kieler H, Ekbom A, et al. Anti-TNF treatment during pregnancy and birth outcomes: A population-based study from Denmark, Finland, and Sweden. Pharmacoepidemiol Drug Saf. 2020;29(3):316-327.	Sweden, Denmark, Finland	Cohort	to study the risk of preterm birth, caesarean section, and small for gestational age after anti-tumor necrosis factor agent treatment in pregnancy	Swedish, Danish, and Finnish medical registers	RA, PsA, AS	adalimumab, certolizumab-pegol, etanercept, golimumab, infliximab, hydroxychloroquine, azathioprine, cyclosporine, sulfasalazine, tacrolimus, methotrexate, leflunomide, mycophenolic acid	preterm birth, caesarean delivery, small for gestational age
3	Bérard A, Sheehy O, Zhao JP, Vinet E, Quach C, Bernatsky S. Chloroquine and Hydroxychloroquine Use During Pregnancy and the Risk of Adverse Pregnancy Outcomes Using Real-World Evidence. Front Pharmacol. 2021;12:722511.	Canada	Case-control	to investigate the safety of chloroquine or hydroxychloroquine use in a large pregnancy cohort using real-world evidence	Quebec claims databases	SLE, RA	hydroxychloroquine	preterm birth, low birth weight, major congenital malformations
4	Bröms G, Haerskjold A, Granath F, Kieler H, Pedersen L, Berglind IA. Effect of Maternal Psoriasis on Pregnancy and Birth Outcomes: A Population-based Cohort Study from	Sweden, Denmark	Cohort	to assess the effect of maternal psoriasis and its severity on adverse pregnancy and	Swedish and Danish medical registers	PsA	adalimumab, certolizumab-pegol, etanercept, golimumab, infliximab, ustekinumab,	gestational diabetes, gestational hypertension, antenatal hemorrhage, venous thromboembolism during pregnancy,

ID	Citation	Country	Study design	Objective(s)	Data sources	Diseases	Medications	APOs
	Denmark and Sweden. Acta Derm Venereol. 2018;98(8):728-734.			birth outcomes using a population-based register data in Sweden and Denmark			abatacept, cyclosporin, methotrexate, leflunomide	preeclampsia, caesarean delivery, preterm birth, low Apgar score, small for gestational age, low birth weight, stillbirth, major congenital malformations
5	Gernaat SAM, Simard JF, Wikström AK, Svenungsson E, Arkema EV. Gestational Diabetes Mellitus Risk in Pregnant Women With Systemic Lupus Erythematosus. J Rheumatol. 2022;49(5):465-469.	Sweden	Cohort	to investigate the risk of gestational diabetes mellitus associated with systemic lupus erythematosus by comparing pregnancies in women with SLE to general population controls	Swedish medical registers	SLE	hydroxychloroquine	gestational diabetes
6	Carman WJ, Accortt NA, Anthony MS, Iles J, Enger C. Pregnancy and infant outcomes including major congenital malformations among women with chronic inflammatory arthritis or psoriasis, with and without etanercept use. Pharmacoepidemiol Drug Saf. 2017;26(9):1109-1118.	United States	Cohort	to quantify prevalence estimates of pregnancy and infant outcomes including major congenital malformations by etanercept exposure among infants born to women with chronic inflammatory arthritis or psoriasis	Optum	RA, PsA, AS	etanercept, adalimumab, certolizumab-pegol, golimumab, infliximab, hydroxychloroquine, azathioprine, methotrexate, leflunomide	stillbirth, spontaneous abortion (miscarriage, ectopic pregnancy, and trophoblastic pregnancy), induced abortion, low birth weight, preterm birth, congenital malformations, derived outcome of having at least one major congenital malformation, maternal infection, hemorrhage in early pregnancy, early or threatened labor
7	Chen YJ, Chang JC, Lai EL, et al. Maternal and perinatal outcomes of pregnancies in systemic lupus erythematosus: A nationwide	Taiwan	Case-control	to explore the risk of pregnancy complications in	Taiwan's National Health	SLE	hydroxychloroquine, azathioprine, cyclosporine	placenta previa and placental abruption, pregnancy-associated hypertension diseases or

ID	Citation	Country	Study design	Objective(s)	Data sources	Diseases	Medications	APOs
	population-based study. Semin Arthritis Rheum. 2020;50(3):451-457.			Asian patients with SLE	Insurance program			preeclampsia, gestational diabetes, intrauterine growth restriction, dystocia, fetal distress, infection of genitourinary tract in pregnancy, ectopic pregnancy, caesarean delivery, preterm birth, low Apgar score, stillbirth, preterm labor, low birth weight, high birth weight
8	Cooper WO, Cheetham TC, Li DK, et al. Brief report: Risk of adverse fetal outcomes associated with immunosuppressive medications for chronic immune-mediated diseases in pregnancy. Arthritis Rheumatol. 2014;66(2):444-450.	United States	Cohort	to assess the risk of adverse fetal outcomes following exposure to individual immunosuppressi ve drugs in pregnant women with chronic immune-mediated diseases	Tennessee Medicaid, Kaiser Permanente Northern California, Kaiser Permanente Southern California	SLE, RA, PsA, AS	adalimumab, etanercept, infliximab, hydroxychloroquine, azathioprine, sulfasalazine, methotrexate, leflunomide	congenital malformations, fetal death, and life-threatening neonatal complications (respiratory failure, seizure, moderate-to-severe jaundice, or sepsis), preterm birth, stillbirth
9	Cleary BJ, Källén B. Early pregnancy azathioprine use and pregnancy outcomes. Birth Defects Res A Clin Mol Teratol. 2009;85(7):647-654.	Sweden	Cohort	to examine the pregnancy outcomes of women exposed to azathioprine in early pregnancy using a population-based medical birth register with medication exposures prospectively	Swedish medical registers	SLE, rheumatic diseases (unspecified)	azathioprine, hydroxychloroquine, sulfasalazine, tacrolimus	preterm birth, stillbirth, subarachnoid hemorrhage, low birth weight, small for gestational age, high birth weight, large for gestational age, congenital malformations

ID	Citation	Country	Study design	Objective(s)	Data sources	Diseases	Medications	APOs
				ascertained early in pregnancy				
10	Huybrechts KF, Bateman BT, Zhu Y, et al. Hydroxychloroquine early in pregnancy and risk of birth defects. Am J Obstet Gynecol. 2021;224(3):290.e1-290.e22.	United States	Cohort	to evaluate the risk of major congenital malformations associated with exposure to hydroxychloroquine during the first trimester of pregnancy, the period of organogenesis	Medicaid, MarketScan	SLE, RA, PsA, AS	hydroxychloroquine	major congenital malformations diagnosed during the first 90 days after delivery and specific malformation types for which there were at least 5 exposed events: oral cleft, cardiac, respiratory, gastrointestinal, genital, urinary, musculoskeletal, and limb defects
11	Bortoluzzi A, Andreoli L, Carrara G, et al. Improved Pregnancy Outcome in Patients With Rheumatoid Arthritis Who Followed an Ideal Clinical Pathway. Arthritis Care Res (Hoboken). 2021;73(2):166-172.	Italy	Cohort	to assess the effect of optimal management of pregnancy on a composite outcome of miscarriage and complicated birth among women with rheumatoid arthritis	Administrative healthcare databases of the Lombardy region in Italy	RA	adalimumab, certolizumab-pegol, etanercept, golimumab, infliximab, abatacept, tocilizumab, rituximab, anakinra, hydroxychloroquine, azathioprine, sulphasalazine, cyclosporine, methotrexate, leflunomide	caesarean delivery, pregnancy loss (stillbirth, miscarriage, ectopic pregnancy), threatened miscarriage, perinatal death
12	Petri M, Daly RP, Pushparajah DS. Healthcare costs of pregnancy in systemic lupus erythematosus: retrospective observational analysis from a US health claims database. J Med Econ. 2015;18(11):967-973.	United States	Cohort	to quantify drug and resource utilization in pregnant women with lupus, as well as the incidence of pregnancy complications in these patients	MarketScan	SLE	hydroxychloroquine methotrexate, mycophenolate	gestational hypertension, preeclampsia, preterm birth, stillbirth, miscarriage, threatened miscarriage, premature labor without delivery

ID	Citation	Country	Study design	Objective(s)	Data sources	Diseases	Medications	APOs
13	Bröms G, Granath F, Ekbom A, et al. Low Risk of Birth Defects for Infants Whose Mothers Are Treated With Anti-Tumor Necrosis Factor Agents During Pregnancy. Clin Gastroenterol Hepatol. 2016;14(2):234-41.e415.	Sweden, Denmark	Cohort	to study the risk of birth defects after anti-TNF treatment in early pregnancy	Swedish and Danish medical registers	RA, PsA, AS	adalimumab, certolizumab-pegol, etanercept, golimumab, infliximab, hydroxychloroquine, azathioprine, sulfasalazine, cyclosporine, tacrolimus, methotrexate mycophenolic acid, mercaptopurine, cyclophosphamide	birth defects, in general and by organ system-specific subgroups, and hospital visits in infants up to one year of age
14	Skorpen CG, Lydersen S, Gilboe IM, et al. Influence of disease activity and medications on offspring birth weight, pre-eclampsia and preterm birth in systemic lupus erythematosus: a population-based study. Ann Rheum Dis. 2018;77(2):264-269.	Norway	Cohort	to explore the possible associations of disease activity and medications with offspring birth weight z-score and the occurrence of pre-eclampsia and preterm birth in women with SLE	Norwegian medical registers	SLE	hydroxychloroquine, azathioprine	preeclampsia, preterm birth, low birth weight, small for gestational age
15	Venne K, Scott S, Bernatsky S, Vinet E. Induced abortions in women with systemic lupus erythematosus. Lupus. 2021;30(3):484-488.	Canada	Nested case-control	to determine the rates of induced abortions in women with SLE compared to women from the general population and assess disease-related predictors of induced	Quebec claims databases	SLE	azathioprine, sulfasalazine, cyclosporine, methotrexate, leflunomide, mycophenolate mofetil, cyclophosphamide	induced abortion

ID	Citation	Country	Study design	Objective(s)	Data sources	Diseases	Medications	APOs
				abortion in women with SLE				
16	Vinet É, Pineau CA, Scott S, Clarke AE, Platt RW, Bernatsky S. Increased congenital heart defects in children born to women with systemic lupus erythematosus: results from the offspring of Systemic Lupus Erythematosus Mothers Registry Study. Circulation. 2015;131(2):149-156.	Canada	Cohort	to determine whether children born to women with SLE have an increased risk of congenital heart defects in comparison with children born to women without SLE	Quebec claims databases	SLE	hydroxychloroquine, azathioprine, methotrexate, mycophenolate mofetil, mycophenolic acid	congenital heart defects, preeclampsia, eclampsia, gestational diabetes, preterm birth, stillbirth, induced abortion, small for gestational age
17	Viktil KK, Engeland A, Furu K. Outcomes after anti-rheumatic drug use before and during pregnancy: a cohort study among 150,000 pregnant women and expectant fathers. Scand J Rheumatol. 2012;41(3):196-201.	Norway	Cohort	to study (i) the drug utilization pattern of anti-rheumatic drugs in pregnant women and expectant fathers and (ii) the association between the use of anti-rheumatic drugs during pregnancy and the risk of congenital malformations	Norwegian medical registers	rheumatic diseases (unspecified)	adalimumab, etanercept, anakinra, hydroxychloroquine, azathioprine, sulfasalazine, methotrexate, leflunomide	congenital malformations, preterm birth, stillbirth, miscarriage, induced abortion, infant death within 28 days of birth
18	Kuriya B, Hernández-Díaz S, Liu J, Bermas BL, Daniel G, Solomon DH. Patterns of medication use during pregnancy in rheumatoid arthritis. Arthritis Care Res (Hoboken). 2011;63(5):721-728.	United States	Cohort	to characterize therapies prescribed during pregnancy to women with RA	Blue Cross, Blue Shield	RA	adalimumab, etanercept, infliximab, rituximab, anakinra, hydroxychloroquine, azathioprine, sulfasalazine, cyclosporine, methotrexate, leflunomide	induced abortion, miscarriage

ID	Citation	Country	Study design	Objective(s)	Data sources	Diseases	Medications	APOs
19	Redeker I, Strangfeld A, Callhoff J, Marschall U, Zink A, Baraliakos X. Maternal and infant outcomes in pregnancies of women with axial spondyloarthritis compared with matched controls: results from nationwide health insurance data. RMD Open. 2022;8(2):e002146.	Germany	Cohort	to investigate pregnancy outcomes in women with axial spondyloarthritis (AxSpA) under different pharmacological treatments in comparison with matched controls	BARMER	AS	adalimumab, certolizumab-pegol, etanercept, golimumab, infliximab, abatacept, tocilizumab, ustekinumab, secukinumab, azathioprine, sulfasalazine, cyclosporine, tacrolimus, methotrexate leflunomide, mycophenolate	stillbirth, induced abortion, ectopic pregnancy, miscarriage, preterm birth, caesarean delivery, preeclampsia, gestational diabetes, small for gestational age, congenital malformations
20	Howren A, Rebić N, Sayre EC, et al. Perinatal exposure to conventional synthetic disease-modifying anti-rheumatic drugs in women with rheumatic disease and neonatal outcomes: a population-based study. Clin Exp Rheumatol. 2020;38(6):1080-1087.	Canada	Cohort	to evaluate the association between perinatal csDMARD use among women with rheumatic diseases and adverse neonatal outcomes, specifically, small-for-gestational-age births and congenital anomalies, which represent leading causes of infant morbidity	British Columbia claims databases	RA, PsA, AS	hydroxychloroquine, azathioprine, sulfasalazine, cyclosporine, methotrexate, leflunomide, mycophenolate mofetil, cyclophosphamide, biologics (unspecified)	small for gestational age, congenital malformations, caesarean delivery
21	Hellgren K, Secher AE, Glintborg B, et al. Pregnancy outcomes in relation to disease activity and anti-rheumatic treatment strategies in women with rheumatoid arthritis: a matched cohort study from Sweden and	Sweden, Denmark	Cohort	to explore the association of maternal RA to pregnancy outcomes, especially	Swedish and Danish medical registers	RA	adalimumab, certolizumab-pegol, etanercept, golimumab, infliximab, rituximab, abatacept,	preterm birth, small for gestational age, large for gestational age, caesarean delivery, stillbirth

ID	Citation	Country	Study design	Objective(s)	Data sources	Diseases	Medications	APOs
	Denmark. Rheumatology (Oxford). 2022;61(9):3711-3722.			preterm birth and small for gestational age, in relation to disease activity and anti-rheumatic treatment before and during pregnancy			tocilizumab, hydroxychloroquine, azathioprine, sulfasalazine, cyclosporine, methotrexate leflunomide, mycophenolate	
22	Remaeus K, Johansson K, Granath F, Stephansson O, Hellgren K. Pregnancy Outcomes in Women With Psoriatic Arthritis in Relation to Presence and Timing of Antirheumatic Treatment. Arthritis Rheumatol. 2022;74(3):486-495.	Sweden	Cohort	to evaluate pregnancy outcomes in relation to antirheumatic treatment before and during pregnancy, as a proxy of disease severity in pregnant women with PsA, compared to those without PsA	Swedish medical registers	PsA	adalimumab, certolizumab-pegol, etanercept, golimumab, infliximab, abatacept, anakinra, tocilizumab, hydroxychloroquine, azathioprine, sulfasalazine, cyclosporine, methotrexate, leflunomide, mycophenolate	preeclampsia, gestational diabetes, gestational hypertension, preterm birth, caesarean delivery, small for gestational age, large for gestational age
23	Jakobsson GL, Stephansson O, Askling J, Jacobsson LT. Pregnancy outcomes in patients with ankylosing spondylitis: a nationwide register study. Ann Rheum Dis. 2016;75(10):1838-1842.	Sweden	Case-control	to study the occurrence of adverse pregnancy outcomes such as pre-eclampsia, preterm birth, low 5 min Apgar score (<7), small-for-gestational-age, large-for-gestational-age, stillbirth, and delivery outcomes such as	Swedish medical registers	AS	adalimumab, certolizumab-pegol, etanercept, golimumab, infliximab, abatacept, anakinra, hydroxychloroquine, azathioprine, sulfasalazine, cyclosporine, methotrexate	preeclampsia, preterm birth, low Apgar score, small for gestational age, large for gestational age, stillbirth, caesarean delivery

ID	Citation	Country	Study design	Objective(s)	Data sources	Diseases	Medications	APOs
				use of C-section and the use of epidural analgesia among women with AS compared with population-based controls				
24	Tsao NW, Hanley GE, Lynd LD, Amiri N, De Vera MA. Risk of congenital anomalies in infants born to women with autoimmune disease using biologics before or during pregnancy: a population-based cohort study. Clin Exp Rheumatol. 2019;37(6):976-982.	Canada	Cohort	to determine the association between perinatal biologic use and congenital anomalies in women with autoimmune disease	British Columbia claims databases	SLE, RA, PsA, AS	adalimumab, certolizumab-pegol, etanercept, golimumab, infliximab, rituximab, abatacept, belimumab, anakinra, tocilizumab, ustekinumab, csDMARDs (unspecified)	congenital malformations at birth and within first year of life, gestational diabetes, gestational hypertension
25	Secher AEP, Granath F, Glintborg B, Rom A, Hetland ML, Hellgren K. Risk of pre-eclampsia and impact of disease activity and antirheumatic treatment in women with rheumatoid arthritis, axial spondylarthritis and psoriatic arthritis: a collaborative matched cohort study from Sweden and Denmark. RMD Open. 2022;8(2):e002445.	Sweden, Denmark	Cohort	to explore the risk of preeclampsia in RA, AxSpA, and PsA, focusing on the impact of treatment and disease activity	Swedish and Danish medical registers	RA, PsA, AS	adalimumab, certolizumab-pegol, etanercept, golimumab, infliximab, rituximab, abatacept, tocilizumab, hydroxychloroquine, azathioprine, sulfasalazine, cyclosporine, methotrexate, leflunomide, mycophenolate	preeclampsia, eclampsia
26	Lee JS, Oh JS, Kim YJ, et al. Reasons for the High Cesarean Delivery Rate among Women with Ankylosing Spondylitis: Using the Korean National Health Insurance Database. J Rheumatol. 2020;47(5):668-673.	South Korea	Case-control	to identify factors associated with cesarean births in women with AS	South Korea's National Health Insurance program	RA, AS	adalimumab, etanercept, golimumab, infliximab, sulfasalazine, methotrexate	caesarean delivery

ID	Citation	Country	Study design	Objective(s)	Data sources	Diseases	Medications	APOs
27	Tsao NW, Sayre EC, Hanley G, et al. Risk of preterm delivery and small-for-gestational-age births in women with autoimmune disease using biologics before or during pregnancy: a population-based cohort study. Ann Rheum Dis. 2018;77(6):869-874.	Canada	Cohort	to assess the risk of preterm delivery and small for gestational age births in women with autoimmune diseases using biologics before or during pregnancy	British Columbia claims databases	SLE, RA, PsA, AS	adalimumab, certolizumab-pegol, etanercept, golimumab, infliximab, rituximab, abatacept, belimumab, anakinra, tocilizumab, ustekinumab	preterm birth, small for gestational age
28	Desai RJ, Bateman BT, Huybrechts KF, et al. Risk of serious infections associated with use of immunosuppressive agents in pregnant women with autoimmune inflammatory conditions: cohort study. BMJ. 2017;356:j895.	United States	Cohort	to compare the risk of serious infections associated with use of systemic steroids, non-biologic agents, or tumor necrosis factor α inhibitors in pregnancy	Medicaid, Optum	SLE, RA, PsA, AS	adalimumab, certolizumab-pegol, etanercept, golimumab, infliximab, hydroxychloroquine, azathioprine, sulfasalazine, cyclosporine, methotrexate, leflunomide, mycophenolate mofetil	maternal infections during pregnancy requiring hospitalization
29	Palmsten K, Hernández-Díaz S, Kuriya B, Solomon DH, Setoguchi S. Use of disease-modifying antirheumatic drugs during pregnancy and risk of preeclampsia. Arthritis Care Res (Hoboken). 2012;64(11):1730-1738.	Canada	Cohort	to describe patterns of DMARD use during pregnancy in a population-based cohort, and to evaluate the association between autoimmune disease, DMARDs, corticosteroids, and NSAIDs and preeclampsia	British Columbia claims databases	SLE, RA	etanercept, infliximab, hydroxychloroquine, azathioprine, sulfasalazine, cyclosporine, methotrexate, leflunomide, mycophenolate mofetil, mercaptopurine, cyclophosphamide	preeclampsia, preterm birth

ID	Citation	Country	Study design	Objective(s)	Data sources	Diseases	Medications	APOs
30	Tsao NW, Lynd LD, Sayre EC, Sadatsafavi M, Hanley G, De Vera MA. Use of biologics during pregnancy and risk of serious infections in the mother and baby: a Canadian population-based cohort study. BMJ Open. 2019;9(2):e023714.	Canada	Cohort	to investigate the association between exposure to biologics during pregnancy and serious infections in mothers and infants	British Columbia claims databases	SLE, RA, PsA, AS	adalimumab, certolizumab-pegol, etanercept, golimumab, infliximab, rituximab, abatacept, belimumab, anakinra, tocilizumab, ustekinumab, hydroxychloroquine, azathioprine, sulfasalazine, cyclosporine, methotrexate, leflunomide, mycophenolate mofetil, mercaptopurine, cyclophosphamide	maternal infections during pregnancy requiring hospitalization, gestational diabetes, gestational hypertension, caesarean delivery
31	Ahn SM, Joo YB, Kim YJ, Bang SY, Lee HS. Pregnancy Outcomes Associated With Biologic Agent Exposure in Patients With Several Rheumatic Diseases and Inflammatory Bowel Diseases. J Korean Med Sci. 2023;38(22):e172.	South Korea	Cohort	to investigate whether biologic agent exposure was associated with an increase in adverse pregnancy outcomes in patients with rheumatic diseases and IBD using the National Health Insurance Service (NHIS) claims database of South Korea	South Korea's National Health Insurance program	RA, PsA, AS	etanercept, infliximab, adalimumab, golimumab, tocilizumab, secukinumab, ixekizumab, ustekinumab, vedolizumab, tofacitinib, baricitinib, rituximab, and abatacept, methotrexate, leflunomide, azathioprine, sulfasalazine, hydroxychloroquine, tacrolimus	spontaneous abortion, induced abortion, stillbirth, ectopic pregnancy, preterm delivery, preeclampsia, eclampsia, gestational diabetes, intrauterine growth restriction, caesarean delivery

ID	Citation	Country	Study design	Objective(s)	Data sources	Diseases	Medications	APOs
32	Elfving P, Kariniemi S, Kautiainen H, et al. Pregnancies in patients with systemic lupus erythematosus during 2000-2018 in Finland: a case-control study. Rheumatol Int. 2024;44(6):1101-1109.	Finland	Case-control	to investigate, how pregnancies proceed in patients with SLE compared to their individually matched population controls	Finnish medical registers	SLE	hydroxychloroquine, azathioprine, cyclosporine, sulfasalazine	gestational hypertension, pre-eclampsia, eclampsia, caesarean delivery, NICU treatment, preterm birth, birth weight, macrosomia, birth length, congenital malformations
33	Morin M, Frisell T, Stephansson O, Hellgren K. Temporal trends in adverse pregnancy outcomes in axial spondyloarthritis in Sweden: a cohort study. Lancet Rheumatol. 2023;5(3):e121-e129.	Sweden	Cohort	to assess the risks of adverse pregnancy outcomes in a large cohort of women with axSpA and investigate how outcomes varied over time and in relation to anti-rheumatic treatment.	Swedish medical registers	AS, PsA, RA, SLE	csDMARDs, TNFi biologics (unspecified)	preterm birth, pre-eclampsia, caesarean delivery, infant infection requiring hospitalization in first year, stillbirth, gestational diabetes, gestational hypertension, SGA, LGA, Apgar score
34	Rector A, Marić I, Chaichian Y, et al. Hydroxychloroquine in Lupus Pregnancy and Risk of Preeclampsia. Arthritis Rheumatol. 2024;76(6):919-927.	United States	Cohort	to investigate whether early-pregnancy HCQ initiation was associated with a lower risk of preeclampsia in pregnant patients with lupus	MarketScan	SLE	azathioprine, sulfasalazine, leflunomide, tacrolimus, colchicine, cyclosporine, hydroxychloroquine, rituximab, ofatumumab, abatacept, belimumab, etanercept, infliximab, adalimumab, certolizumab-pegol, golimumab, anakinra,	pre-eclampsia, preterm birth, gestational diabetes, gestational hypertension

ID	Citation	Country	Study design	Objective(s)	Data sources	Diseases	Medications	APOs
							ustekinumab, tocilizumab, secukinumab	
35	Simard JF, Liu EF, Rector A, et al. Hydroxychloroquine and Pre-eclampsia in a Diverse Cohort of Women With Systemic Lupus Erythematosus. Arthritis Care Res (Hoboken). Published online June 26, 2024.	United States	Cohort	to test the hypothesis that HCQ treatment during pregnancy reduces the risk of pre-eclampsia or eclampsia among pregnant patients with SLE in a diverse patient cohort in a large integrated health system	Kaiser Permanente Northern California	SLE	hydroxychloroquine, azathioprine, belimumab, rituximab, methotrexate, mycophenolate	preterm birth, pre-eclampsia, eclampsia, gestational hypertension
36	Song YJ, Cho SK, Jung YS, et al. Medication utilisation trends during pregnancy and factors influencing adverse pregnancy outcomes in patients with rheumatoid arthritis. RMD Open. 2024;10(1):e003739.	South Korea	Cohort	to investigate medication utilisation patterns during pregnancy and identified factors associated with adverse pregnancy outcomes in patients with RA in Korea	South Korea's National Health Insurance program	RA	methotrexate, leflunomide, hydroxychloroquine, sulfasalazine, tacrolimus, bDMARDs (unspecified)	abortion, stillbirth
37	Zanetti A, Zambon A, Scirè CA, Bortoluzzi A. Impact of rheumatoid arthritis and methotrexate on pregnancy outcomes: retrospective cohort study of the Italian Society for Rheumatology. RMD Open. 2022;8(2):e002412.	Italy	Cohort	to evaluate the impact of rheumatoid arthritis and methotrexate on the probability of becoming pregnant, pregnancy losses, elective termination of	Administrative healthcare databases of the Lombardy region in Italy	RA	methotrexate, leflunomide, cyclosporine, hydroxychloroquine, sulfasalazine, azathioprine	spontaneous abortion, stillbirth, elective termination, congenital malformations

ID	Citation	Country	Study design	Objective(s)	Data sources	Diseases	Medications	APOs
				pregnancy and congenital malformations				
38	Chock EY, Liew Z, Pedersen LH, Thunbo MO. Fetal Growth Associated with Maternal Rheumatoid Arthritis and Juvenile Idiopathic Arthritis. medRxiv. 2024;doi:https://dx.doi.org/10.1101/2024.02.29.24303573	Denmark	Cohort	to evaluate fetal growth among patients with RA/JIA by comparing fetal growth indicators of offspring born to this population, compared to individuals without RA/JIA	Danish medical registers	RA	hydroxychloroquine, sulfasalazine	fetal and birth weight, head circumference, SGA, preterm birth, gestational hypertension, pre-eclampsia, eclampsia, gestational diabetes, stillbirth, caesarean delivery
