# Supplementary material for: Use of healthcare administrative claims data in observational studies of antirheumatic drug effects on pregnancy outcomes: A scoping review
Source: PLoS One. 2025 Mar 31;20(3):e0319703. doi: 10.1371/journal.pone.0319703 (PMC11957274; doi:10.1371/journal.pone.0319703)
Supplement: S2 Table — (PDF) [file pone.0319703.s002.pdf]

S2 Table.

DMARDs	n (%)	Citations
**csDMARDs**		
Hydroxychloroquine	30 (78.9)	2, 3, 5, 6, 7, 8, 9, 10, 11, 12, 13, 14, 16, 17, 18, 20, 21, 22, 23, 25, 28, 29, 30, 31, 32, 34, 35, 36, 37, 38
Azathioprine	26 (68.4)	2, 6, 7, 8, 9, 11, 13, 14, 15, 16, 17, 18, 19, 20, 21, 22, 23, 25, 28, 29, 30, 31, 32, 34, 35, 37
Sulfasalazine	24 (63.2)	2, 8, 9, 11, 13, 15, 17, 18, 19, 20, 21, 22, 23, 25, 26, 28, 29, 30, 31, 32, 34, 36, 37, 38
Cyclosporine	19 (50.0)	2, 4, 7, 11, 13, 15, 18, 19, 20, 21, 22, 23, 25, 28, 29, 30, 32, 34, 37
Tacrolimus	7 (18.4)	2, 9, 13, 19, 31, 34, 36
Colchicine	1 (2.6)	34
Methotrexate	25 (65.8)	2, 4, 6, 8, 11, 12, 13, 15, 16, 17, 18, 19, 20, 21, 22, 23, 25, 26, 28, 29, 30, 31, 35, 36, 37
Leflunomide	20 (52.6)	2, 4, 6, 8, 11, 15, 17, 18, 19, 20, 21, 22, 25, 28, 29, 30, 31, 34, 36, 37
Mycophenolic acid / mycophenolate mofetil	14 (36.8)	2, 12, 13, 15, 16, 19, 20, 21, 22, 25, 28, 29, 30, 35
Cyclophosphamide	5 (13.2)	13, 15, 20, 29, 30
Unspecified	4 (10.5)	10, 24, 27, 33
**bDMARDs**		
Etanercept	22 (57.9)	1, 2, 4, 6, 8, 11, 13, 17, 18, 19, 21, 22, 23, 24, 25, 26, 27, 28, 29, 30, 31, 34
Adalimumab	21 (55.3)	1, 2, 4, 6, 8, 11, 13, 17, 18, 19, 21, 22, 23, 24, 25, 26, 27, 28, 30, 31, 34
Infliximab	21 (55.3)	1, 2, 4, 6, 8, 11, 13, 18, 19, 21, 22, 23, 24, 25, 26, 27, 28, 29, 30, 31, 34
Golimumab	18 (47.4)	1, 2, 4, 6, 11, 13, 19, 21, 22, 23, 24, 25, 26, 27, 28, 30, 31, 34
Certolizumab-pegol	15 (39.5)	2, 4, 6, 11, 13, 19, 21, 22, 23, 24, 25, 27, 28, 30, 34
Abatacept	12 (31.6)	4, 11, 19, 21, 22, 23, 24, 25, 27, 30, 31, 34
Tocilizumab	10 (26.3)	11, 19, 21, 22, 24, 25, 27, 30, 31, 34

DMARDs	n (%)	Citations
Rituximab	10 (26.3)	11, 18, 21, 24, 25, 27, 30, 31, 34, 35
Anakinra	9 (23.7)	11, 17, 18, 22, 23, 24, 27, 30, 34
Ustekinumab	7 (18.4)	4, 19, 24, 27, 30, 31, 34
Belimumab	5 (13.2)	24, 27, 30, 34, 35
Secukinumab	3 (7.9)	19, 31, 34
Ixekizumab	1 (2.6)	31
Ofatumamab	1 (2.6)	34
Vedolizumab	1 (2.6)	31
Unspecified	4 (10.5)	10, 20, 33, 36
