# Supplementary material for: Use of healthcare administrative claims data in observational studies of antirheumatic drug effects on pregnancy outcomes: A scoping review
Source: PLoS One. 2025 Mar 31;20(3):e0319703. doi: 10.1371/journal.pone.0319703 (PMC11957274; doi:10.1371/journal.pone.0319703)
Supplement: S3 Table — (PDF) [file pone.0319703.s003.pdf]

S3 Table.

APOs	n (%)	Citations
Preterm birth (spontaneous or healthcare provider-initiated)	25 (65.8)	1, 2, 3, 4, 6, 7, 8, 9, 12, 14, 16, 17, 19, 21 ,22, 23, 26, 27, 29, 31, 32, 33, 34, 35, 38
Preeclampsia	17 (44.7)	4, 7, 12, 14, 16, 19, 22, 23, 25, 26, 29, 31, 32, 33, 34, 35, 38
Stillbirth	17 (44.7)	4, 6, 7, 8, 9, 11, 12, 16, 17, 19, 21, 23, 31, 33, 36, 37, 38
Caesarean delivery (elective or emergency)	16 (42.1)	1, 2, 4, 7, 11, 19, 20, 21, 22, 23, 26, 30, 31, 32, 33, 38
Congenital anomalies	14 (36.8)	3, 4, 6, 8, 9, 10, 13, 16, 17, 19, 20, 24, 32, 37
Small-for-gestational age	13 (34.2)	2, 4, 9, 14, 16, 19, 20, 21, 22, 23, 27, 33, 38
Gestational diabetes	12 (31.6)	4, 5, 7, 16, 19, 22, 24, 30, 31, 33, 34, 38
Gestational hypertension	11 (28.9)	4, 7, 12, 22, 24, 30, 32, 33, 34, 35, 38
Elective termination / induced abortion	10 (26.3)	6, 15, 16, 17, 18, 19, 31, 34, 36, 37
Miscarriage / spontaneous abortion	10 (26.3)	6, 11, 12, 17, 18, 19, 31, 34, 36, 37
Low birth weight	9 (23.7)	1, 3, 4, 6, 7, 9, 14, 32, 38
Eclampsia	7 (18.4)	7, 16, 25, 31, 32, 35, 38
Maternal and neonatal infections	6 (15.8)	6, 7, 8, 28, 30, 33
Molar / ectopic pregnancy	6 (15.8)	6, 7, 11, 19, 31, 34
Large for gestational age	5 (13.2)	9, 21, 22, 23, 33
Low Apgar score	5 (13.2)	1, 4, 7, 23, 33
Complications during labor	4 (10.5)	6, 7, 8, 12
High birth weight	4 (10.5)	1, 7, 9, 32

APOs	n (%)	Citations
Hemorrhage	3 (7.9)	4, 6, 9
Fetal growth restriction	2 (5.3)	7, 31
Threatened miscarriage	2 (5.3)	11, 12
Infant death within 28 days of birth	1 (2.6)	17
NICU treatment	1 (2.6)	32
Placenta previa and placental abruption	1 (2.6)	7
