# Supplementary material for: Use of healthcare administrative claims data in observational studies of antirheumatic drug effects on pregnancy outcomes: A scoping review
Source: PLoS One. 2025 Mar 31;20(3):e0319703. doi: 10.1371/journal.pone.0319703 (PMC11957274; doi:10.1371/journal.pone.0319703)
Supplement: S4 Table — (PDF) [file pone.0319703.s004.pdf]

S4 Table.

Citation	ICD-9 and/or ICD-10 codes
Preterm birth	
19	ICD-10: P07.2, P07.3
29	ICD-9: 644.0x, 644.2x, 765.x<br><br>ICD-10: P05.x, P07.x, O60.1
31	ICD-10: O60, O60.1, O60.3
Preeclampsia	
4	ICD-10: O11, O14-O15
7	ICD-9: 642, 760.0
19	ICD-10: O14, O15
22	ICD-10: O14, O15
23	ICD-10: O14-O15
25	ICD-10: O14.0-O15.9
29	ICD-9: 642.4x, 642.5x, 642.6x, 642.7x<br><br>ICD-10: O11.x, O14.x, O15.x
31	ICD-10: O11, O13-16
32	ICD-10: O14, O15
33	ICD-10: O14, O15
34	ICD-9: 642.40-642.74<br><br>ICD-10: O11, O14-O15
35	ICD-9: 642.4, 642.40, 642.41, 642.42, 642.43, 642.44, 642.5, 642.50, 642.51, 642.52, 642.53, 642.54, 642.6, 642.60, 642.61, 642.62, 642.63, 642.64, 642.7<br><br>ICD-10: O14, O14.0, O14.00, O14.02, O14.03, O14.04, O14.05, O14.1, O14.10, O14.12, O14.13, O14.14, O14.15, O14.2, O14.20, O14.22, O14.23, O14.24, O14.25, O14.9, O14.90, O14.92, O14.93, O14.94, O14.95, O15, O15.0, O15.00, O15.02, O15.03, O15.1, O15.2, O15.9
Stillbirth	
11	ICD-9: V27.1, V27.3, V27.4, V27.6, V27.7
19	ICD-10: P95, Z37.1, Z37.4, Z37.7
31	ICD-10: O36.4
36	ICD-10: Z37.1, Z37.3, Z37.4, Z37.6, Z37.7, O36.4
37	ICD-9: V270-V277
Caesarean delivery	
1	ICD-10: O82.0, O82.1
19	ICD-10: O82
31	ICD-10: O82, O84.2

Congenital anomalies	
3	ICD9: 740.0-742.9, 743.0-744.9 (excluding 743.6, 743.8, 744.1-744.9), 745.0-747.9 (excluding 747.0, 747.5), 748.0-748.9 (excluding 748.2, 748.3), 749.0-749.2, 750.0-750.9 (excluding 750.0, 750.1, 750.2, 750.5, 750.6, 751.0, 751.5), 752.0-752.9 (excluding 752.4, 752.5, 752.8), 753.0-753.9 (excluding 753.6), 754.0-756.9 (excluding 754.0, 754.1, 754.7, 754.8, 756.0, 756.2), 757.0-757.9 (excluding 757.2-757.6, 757.8), 758.0-758.8 (excluding 758.4), 758.9, 759.0-759.9 (excluding 759.9)<br><br>ICD10: Q00.0-Q07.9, Q10.0-Q18.9 (excluding Q10.0-Q10.6, Q13.0, Q13.2, Q13.5, Q15.8, Q17.0-Q17.5, Q17.8, Q17.9, Q18.0-Q18.9), Q20.0-Q28.9 (excluding Q25.0, Q27.0), Q30.0-Q34.9 (excluding Q30.2, Q30.8, Q31-Q32, Q33.1), Q35.0-Q37.9 (excluding Q35.7), Q38.0-Q45.9 (excluding Q38.1-Q38.6, Q40.0, Q40.1, Q43.0, Q43.4-Q43.9), Q50.0-Q56.9 (excluding Q52.2-Q52.8, Q53, Q54.4, Q55.1, Q55.2, Q55.6, Q55.8, Q55.9), Q60.0-Q64.9 (excluding Q61.0, Q62.7, Q63.3, Q64.2, Q64.3), Q65.0-Q79.9 (excluding Q65.3-Q65.6, Q66.2, Q66.3, Q66.5-Q66.9, Q67.0-Q67.4, Q67.6-Q67.8, Q68.0. Q68.1, Q68.3-Q68.8, Q70.3, Q74.1, Q75.0, Q75.2, Q75.3, Q75.8, Q76.0, Q76.5, Q79.5, Q79.8), Q80.0-Q84.9 (excluding Q81, Q82.1- Q82.8, Q83.2, Q83.3, Q83.8, Q84.1- Q84.6, Q84.8), Q90.0-Q99.2 (excluding Q95.0, Q95.1), Q85.0-Q89.9, Q99.8, Q99.9 (excluding Q89.9)
4	ICD-10: Q00-Q99
6	ICD-9: 758.xx-758.9x, 745.xx-747.xx, 740.xx-742.xx, 744.xx-744.9x, 743.xx-743.9x, 750.xx-751.9x, 752.xx-753.9x, 754.xx-756.9x, 749.xx-749.9x, 748.xx-748.9x, 757.xx-757.9x, 759.xx-759.9x, 658.81
9	ICD-9: 740-759<br><br>ICD-10: codes starting with a Q
10	ICD-9: 745.0x, 745.1x, 745.2x, 745.3x, 745.4x, 745.5x and not preterm, 745.6x, 746.00, 746.01, 746.09, 746.1x, 746.2x, 746.83, 747.3x and not preterm, 746.02 and not preterm, 747.1x, 747.2x, 746.3x, 746.5x, 746.7x, 746.81, 746.82, 746.84, 747.0x and not preterm, 416.0x or 747.83 and not preterm, 747.4x, 745.7x, 745.8x, 746.8 (excluded if only 746.86), 746.85-746.87, 746.89, 745, 745.9, 746, 746.9x (excluded if only 746.99), 747, 740.xx - 742.xx, 742.1x, 742.3x, 742.2x, 741.xx, 756.17, 740.0x, 740.2x, 742.0x, 754.50, 754.51, 754.59, 754.60, 754.62, 754.69, 754.70, 754.71, 754.79, 756.73 if coded after October 2009, 749.0x, 749.1x, 749.2x, 743.xx (excluded if only 743.6x and 743.8x), 744.xx (excluded if only 744.1x, 744.21, 744.29, and 744.4x-744.9x), 747.6x-747.9x (excluded if only 747.83), 748.xx (excluded if only 748.1x), 750.xx-751.xx (excluded if only 750.0x, 750.1x, 750.50, 751.0x), 752.xx (excluded if only 752.42, 752.52; excluded 752.5x if preterm), 753.xx (excluded if only 753.7x), 754.xx, 756.xx (excluded if only 754.3x, 754.81, 754.82, 756.2x), 755.xx (excluded if only 755.65), 757.xx, 759.xx (excluded if only 757.2-757.6, 759.81-759.83)
13	ICD-10: Q00-Q007, Q11-Q16, Q100, Q104, Q106, Q107, Q178, Q183, Q187, Q188 (excluding Q135), Q20-Q26 (excluding Q250 (included for term birth)), Q30-Q34 (excluding Q314, Q320), Q35-Q37, Q41-Q45, Q402-Q409, Q38, Q39, Q790, Q792, Q793, Q795 (excluding Q381, Q382, Q3850, Q4021, Q4381, Q4382), Q50-Q56 (excluding Q523, Q525, Q53), Q60–Q64, Q794 (excluding Q627, Q633), Q650-Q652, Q658-Q660, Q681, Q682, Q688, Q69-Q74 (excluding Q6821), Q750, Q751, Q754-Q759, Q761-Q764, Q766-Q769, Q77, Q78, Q796-Q799, Q90-Q99 (excluding Q936), Q80-Q87, Q27-Q28, Q89, Q936 (excluding Q270, Q825, Q8280, Q833, Q845, Q899)
16	ICD-9: 745, 746, 7471-7474, 7454, 7455, 746.0-746.6<br><br>ICD-10: Q20-Q26 (excluding Q24.6, Q25.0), Q210, Q211, Q22, Q23
17	ICD-10: Q20-26, Q50-52, Q54-56, Q753, Q35-37, Q65-66, Q74, Q21, Q54, Q375, 379
19	ICD10: Q00-Q07 (excluding Q0461, Q0782), Q10-Q15 (excluding Q101-Q103, Q105, Q135), Q16-Q18 (excluding Q170-Q175, Q179, Q180-Q182, Q184-Q187, Q1880, Q189), Q20-Q26 (excluding Q211, Q250 if preterm, Q2541, Q256 if preterm, Q261), Q300, Q32-Q34 (excluding Q320, Q331), Q35-Q37, Q38-Q45 (excluding Q381, Q382, Q3850, Q400, Q401, Q4021, Q430, Q4320, Q4381, Q4382), Q790, Q792, Q793, Q795, Q50-Q52, Q54-Q56 (excluding Q523, Q525, Q527, Q5520, Q5521, Q60-Q64 (excluding Q610, Q627, Q633), Q794, Q65-Q74 (excluding Q653-Q656, Q658, Q659, Q661-Q669, Q670-Q678, Q680, Q6810, Q6821, Q683-Q685, Q7400), D821, P350, P351, P371, Q0435, Q206, Q240, Q3381, Q411, Q412, Q418,

	Q4471, Q6190, Q710, Q712, Q713, Q720, Q722, Q723, Q730, Q7402, Q7484, Q750, Q751, Q754, Q7581, Q77, Q7800, Q782-Q788, Q793, Q795, Q7980, Q7982, Q80-Q82, Q86, Q87, Q890, Q893, Q894, Q936 (excluding Q825, Q8280, Q8703, Q8704, Q8708, Q8724), Q90-Q92, Q93, Q96-Q99 (excluding Q936)
30	ICD-9: 740-759 (excluding 758, 759.81-83)<br><br>ICD-10: Q00-Q89
32	ICD-10: Q10-18, Q20-28, Q35-37, Q38-45, Q50-56, Q60-64, Q65-79, Q80-89, Q90-99
37	ICD-9: 740-759
